# Liposomal bupivacaine versus standard periarticular injections in total hip and knee arthroplasty: a prospective, randomized non-inferiority trial

**DOI:** 10.1051/sicotj/2025012

**Published:** 2025-03-13

**Authors:** Joseph Bowen, Joshua P. Rainey, Jonathan Linthicum, Brenna E. Blackburn, Lucas A. Anderson

**Affiliations:** 1 Kootenai Health, 2003 Kootenai Health Way Coeur d’Alene Idaho 83814 USA; 2 Department of Orthopaedic Surgery, University of Utah 590 Wakara Way Salt Lake City UT 84108 USA

**Keywords:** Liposomal bupivacaine, Total hip arthroplasty, Total knee arthroplasty, Opioid

## Abstract

*Introduction*: Numerous multimodal pain protocols have been developed to optimize pain control, reduce narcotics consumption, and shorten the length of stay after total hip and knee arthroplasty (THA/TKA). Liposomal bupivacaine (LB) has been postulated to reduce narcotic requirements after arthroplasty but is not without additional cost. The aim of this study was to determine if the addition of LB to our standard periarticular injection would improve postoperative pain and shorten the length of stay in patients undergoing TKA or THA. *Methods*: We performed a prospective randomized, blinded non-inferiority study of patients undergoing THA and TKA. Patients were randomized to a periarticular injection with and without LB. There were 118 hips and 64 knees included in the study with no demographic differences between groups*.* Post-operative pain management was performed by a second provider who was blinded to the patient’s experimental group designation. *Results*: Cost analysis determined that LB increased cost by $305 dollars per patient when accounting for the cost of injections as well as intravenous and oral pain medications. LB led to a minor reduction in narcotic use in THA patients (equivalent to a single 10 mg oxycodone dose), but this difference may lack clinical relevance. No significant benefits were observed in TKA patients. No difference was identified in self-reported pain scores or lengths of hospital stay. *Discussion*: The addition of LB did not significantly reduce narcotic consumption in patients undergoing TKA, while the cost of LB is prohibitive and should be considered an area of potential cost savings by surgeons and hospitals. The minor reduction in narcotic use in patients undergoing THA likely lacks clinical significance.

## Introduction

Perioperative pain control is a fundamental part of patient satisfaction after total hip and knee arthroplasty (THA/TKA). Current pain management protocols use several different mechanisms simultaneously and are termed “multimodal”, and have demonstrated improvement in pain control after surgery while minimizing side effects [1]. Advancement in the understanding and practice of multimodal pain management is one of the prime reasons for shorter hospital stays [2].

A key component of the multimodal approach to pain management in THA and TKA is the intraoperative surgical site injection of a local anesthetic or drug cocktail, a periarticular injection (PAI), with several described formulations in the literature [3–5]. Our institution has long utilized a formulation, which has been anecdotally associated with a reduction of narcotic use and length of stay (LOS). In recent years, an extended-release bupivacaine called liposomal bupivacaine (LB) was thought to extend the effective half-life of the anesthetic agent and thereby prolong post-operative anesthesia [6]. With improved pain control, it was believed that a corresponding reduction in narcotics and their associated complications, such as respiratory depression and constipation, would thereby expedite discharge and reduce rehospitalization [7]. The use of LB in THA and TKA has been rapidly adopted at many centers and has become an integral part of the “same day total joint” movement [8]. Simultaneously, with the expansion of bundled payments and emphasis on exploding healthcare costs, there has been an effort to cut unnecessary costs and reevaluate expenses that may or not contribute to “success” in arthroplasty [9].

In a recent systematic review of 63 randomized controlled trials evaluating LB in postoperative pain management and opioid consumption, the authors concluded that the majority of studies did not support the efficacy of LB [10]. The authors additionally noted underreporting trial results and bias due to underlying financial relationships. The aim of this non-industry-funded study was to determine whether the addition of LB to our standard PAI would improve pain control or reduce LOS in patients undergoing TJA. Secondary goals were (1) to quantify in oral morphine equivalents the added benefit of LB in addition to, (2) analyzing the added cost of LB to our standard PAI. Our hypotheses were that the addition of LB to our standard PAI would (1) not significantly reduce narcotic intake in morphine equivalents and (2) not significantly shorten LOS in patients undergoing primary TJA.

## Material and methods

Institutional review board approval was obtained to conduct this prospective, randomized, double-blinded study comparing PAI combination with LB suspension (Treatment A) versus PAI without the addition of LB (Treatment B) for patients undergoing TKAH or THA (Table 1). The goal of this non-inferiority trial was to quantify the benefit provided by the addition of bupivacaine LB suspension to the PAI. A non-inferiority design was conducted given the heterogenous data that exists on LB in the treatment of patients undergoing THA and TKA and given the current success of PAIs in TKA and THA [10].

**Table 1:** Periarticular injection assignments and costs.

Treatment A	Treatment B
20 mL Liposomal Bupivacaine	
49.25 mL 5 mg/mL Bupivacaine	49.25 mL 5 mg/mL Ropivacaine
1.0 mL 1:1000 Epinephrine	1.0 mL 1:1000 Epinephrine
0.8 mL Clonidine 100 mcg/mL	0.8 mL Clonidine 100 mcg/mL
13.7 mL NaCl 0.9%	13.7 mL NaCl 0.9%
1.0 mL Ketorolac (15 – 30 mg)^*^	1.0 mL Ketorolac (15 – 30 mg)*

* May be held for patients with compromised renal function.

For the THA cohort, we sought to enroll all patients of one surgeon from the dates January 25, 2016, to October 23, 2017, who met the inclusion criteria. For the TKA cohort we attempted to enroll all patients of three surgeons at a single facility from January 25, 2017, to August 9, 2018. The randomization schedule was set up using a routine alternating treatment assignment between each patient in a 1:1 pattern. Patients undergoing THA versus TKA were randomized separately, given the relative time interruption nature of this study.

Inclusion criteria included patients between 20 and 85 years of age undergoing THA by the lead author for a preoperative diagnosis of hip osteoarthritis, inflammatory arthritis, post-traumatic arthritis, and avascular necrosis of the hip or those undergoing TKA by three surgeons for knee osteoarthritis, inflammatory arthritis, post-traumatic arthritis, or osteonecrosis. Exclusion criteria included the following: multiple daily doses of long-acting narcotics (such as Oxycontin, MS Contin, Fentanyl patches, etc.); prior hip or knee arthroplasty surgery on the same joint (current revision arthroplasty); an intraoperative surgical complication (i.e.: femoral fracture with implant insertion, nerve or artery injury, etc.); allergy or other medical contraindications to the joint injection; the inability of the patient to provide informed consent.

### Surgical technique

All THAs were performed with an anterolateral approach by one surgeon (JMB) and used uncemented components. All TKAs were performed by one of the three surgeon authors (JMB, LAA, JL) and were performed with standard technique through a medial parapatellar arthrotomy with cemented components. Post-operative pain management was performed by a set of nurse practitioners blinded to patient randomization.

Short-acting spinal blocks were utilized whenever possible. The general anesthetic was used when patients declined spinal or the spinal failed to provide adequate anesthesia. We did not prospectively collect anesthesia data.

### Perioperative multimodal pain protocol

Unless there was a noted allergy or medical contraindication, every patient received the following preoperative medications by mouth (PO) 30–60 min prior to surgery: celecoxib 200 mg, acetaminophen 1000 mg, dexamethasone 4 mg, and tramadol 50 mg, ondansetron 4 mg and pantoprazole 40 mg. Tranexamic acid 1 gm IV was used prior to incision as well as a second IV dose 3 h later.

### Postoperative multimodal pain protocol

Postoperative pain meds were as follows: Acetaminophen 1000 mg every 8 h PO scheduled, tramadol 50 mg every 6 h scheduled PO, oxycodone 5–10 mg every 4 h as-needed PO, Dilaudid 0.5–2 mg IV every 2 h as-needed. Aspirin 325 mg was used twice daily PO for deep vein thrombosis (DVT) prophylaxis for six weeks unless patients had documented prior DVTs, active cancer, or other thrombotic risk factors.

### Injection contents and technique

Injections utilized in this study included our institution’s standard PAI with PB for Treatment A versus Treatment B (PAI without LB) (Table 1). Treatment B solutions contained ropivacaine rather than bupivacaine, a well-tolerated regional anesthetic with an efficacy broadly like that of bupivacaine [11, 12]. The other drugs contained in the periarticular injection included: ketorolac, a non-steroidal anti-inflammatory drug (NSAID); clonidine, an alpha-2 adrenergic agonist; and epinephrine, an alpha and beta-adrenergic agonist. Epinephrine was added to produce local vasoconstriction prolong local medication concentrations and potentiate analgesia. This medication cocktail is believed to have a synergistic effect for improved pain control [4]. The lead author and surgeons were not blinded to the treatment due to the contrasting appearances of the two injections in the syringes. However, the postoperative providers (nursing team, anesthesia team, and orthopedic advanced practice providers) were blinded to the randomized assignment group. The advanced practice providers who routinely round on postoperative patients and adjust pain regimens per relative pain control were blinded to the assignment group. Although the lack of blinding of the participating surgeons could have led to injection bias, we believe this was fairly minimal. Importantly, the postoperative providers were blinded to the randomized assignment group, and these were the individuals managing pain postoperatively.

### Outcomes

As a non-inferiority trial, the primary outcome measured was the difference in average pain scores during the hospital stay. Pain was assessed using the visual analog scale pain score from 0 to 10 scale with 0 being no pain to 10, which is the worst pain the patient has experienced. Pain, along with the patient’s pain goal, was assessed every 4 h from post-anesthesia care unit (PACU) arrival to discharge. Based on previously published data, a minimum of 126 patients (63 in each group) were required for a power of 0.8 at *α* = 0.05 to detect a one-point difference in Visual Analog Scores for pain [13]. Mean pain scores were compared between the two treatment cohorts and stratified by TKA/THA within each treatment cohort.

Secondary outcomes included opioid use, LOS, and a cost analysis. Opioid use was collected from PACU to discharge, tabulated, and converted to oral morphine milligram equivalents (MME), and compared between the two cohorts. A cost analysis of Treatment A versus B included the cost of the standard PAI, the addition of LB, and the cost of IV and oral medications used by the two groups.

### Statistical analysis

A comparison of patient characteristics between patients in Treatment A and Treatment B was performed using chi-squared and t-tests. T-tests were used to analyze differences in average pain, MME, and length of stay. A linear, mixed-effects model was used to assess the differences in pain scores and opioid use. Stratification by procedure type (TKA or THA) was also performed.

## Results

Of the 188 patients enrolled in this prospective study, three were determined to be ineligible, and three canceled surgery after enrollment, leaving 182 patients who completed the study (92 in Treatment A and 90 in Treatment B). There were no differences in sex, age, or body mass index (BMI) between groups (Table 2).

**Table 2:** Patient characteristics. Continuous variables are summarized by the mean (SD) and accompanied by boxplots.

	Total (*n* = 182)	Treatment A (*n* = 92)	Treatment B (*n* = 90)	
	*n* (%)	*n* (%)	*n* (%)	*p*-Value
Sex				
Female	112 (61.5)	57 (62.0)	55 (61.1)	0.9067
Male	70 (38.5)	35 (38.0)	35 (38.9)	
Joint				
Knee	64 (35.2)	32 (34.8)	32 (35.6)	0.9131
Hip	58 (64.4)	60 (65.2)	58 (64.4)	

	Mean (SD)	Mean (SD)	Mean (SD)	*p*-Value

Age	65.6 (10.2)	66.2 (9.6)	64.9 (10.8)	0.3734
BMI	31.0 (6.3)	30.3 (6.1)	31.7 (6.4)	0.1471
Weight (kg)	89.7 (20.6)	88.5 (19.8)	90.9 (21.3)	0.419

**Figure 1:**
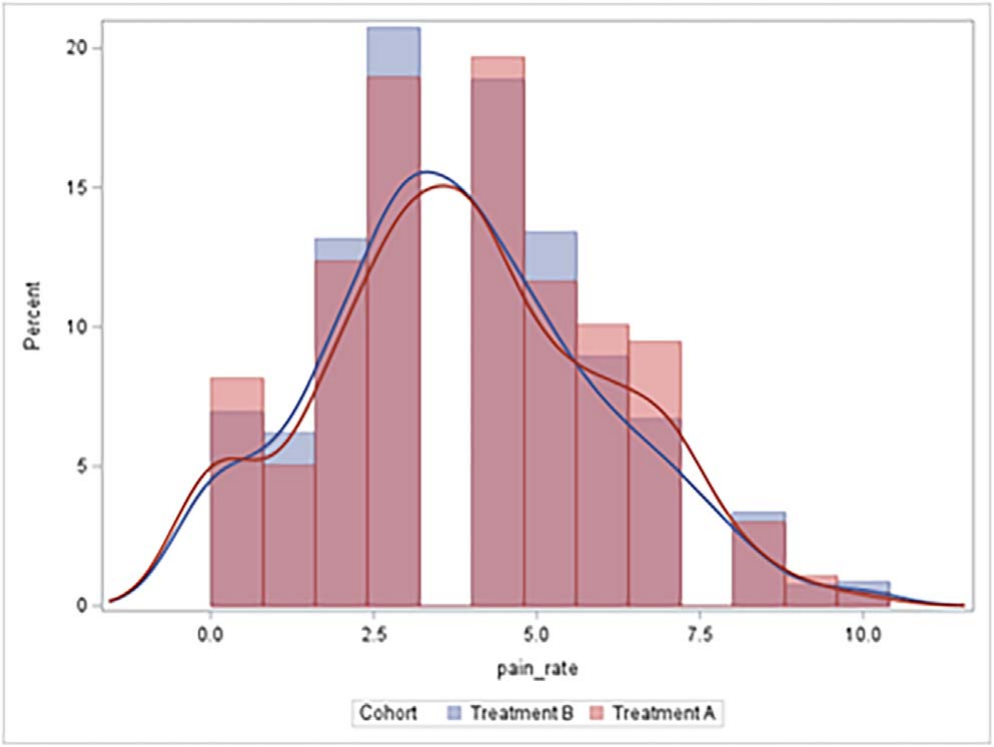
Distribution of pain scores over time duration of the patients’ stays.

Figure 1 displays the distribution of pain scores over the duration of the hospital stay. There was no significant difference in the average pain scores during the first 24 h of surgery [3.8 (SD = 2.1) in Treatment A versus 3.6 (SD = 2.1) in Treatment B, *p* = 0.2560] or during the entire hospital stay [3.7 (SD = 1.6) vs. 3.5 (SD = 1.3), *p* = 0.4898] (Table 3). Although these findings were not statistically significant, a 0.2-point reduction in pain certainly lacks clinical significance. There was also no significant difference in pain goals between these two groups. After adjustment for age, BMI, and sex, there was no difference in pain in the repeated measures linear mixed effects model (*β* = 0.24, *p* = 0.2345).

**Table 3:** Outcomes by treatment.

	Treatment A (*n* = 92)	Treatment B (*n* = 90)	
Outcomes	Mean (SD)	Mean (SD)	*p*-Value
Knees and hips			
Average pain in first 24 h	3.8 (2.1)	2.6 (2.1)	0.2560
Average pain duration of hospital stay	3.7 (1.6)	3.5 (1.3)	0.4898
Average pain goal	3.7 (0.8)	3.5 (0.7)	0.2163
Total MME	161.8 (119.3)	156.4 (180.6)	0.8126
MME/hour of stay	3.6 (2.4)	3.2 (2.1)	0.2654
Length of stay (hours)	45.1 (19.1)	45.2 (27.2)	0.9915
Knees only			
Average pain in first 24 h	3.5 (2.4)	3.2 (1.9)	0.2313
Average pain duration of hospital stay	3.6 (1.9)	3.3 (1.2)	0.4825
Average pain goal	3.8 (0.96)	3.7 (0.6)	0.6319
Total MME	140.3 (115.1)	114.4 (97.3)	0.3349
MME/hr of stay	3.4 (2.2)	2.9 (1.9)	0.2620
Length of stay (hours)	38.9 (18.4)	39.6 (20.6)	0.9006
Hips only			
Average pain in first 24 h	3.9 (2.0)	2.8 (2.2)	0.6641
Average pain duration of hospital stay	3.7 (1.4)	3.7 (1.4)	0.7643
Average pain goal	3.6 (0.7)	3.5 (0.7)	0.2227
Total MME	173.2 (120.8)	179.5 (210.3)	0.8430
MME/hour of stay	3.7 (2.5)	3.5 (2.2)	0.5547
Length of stay (hours)	48.4 (18.8)	48.2 (29.9)	0.96713

**Figure 2:**
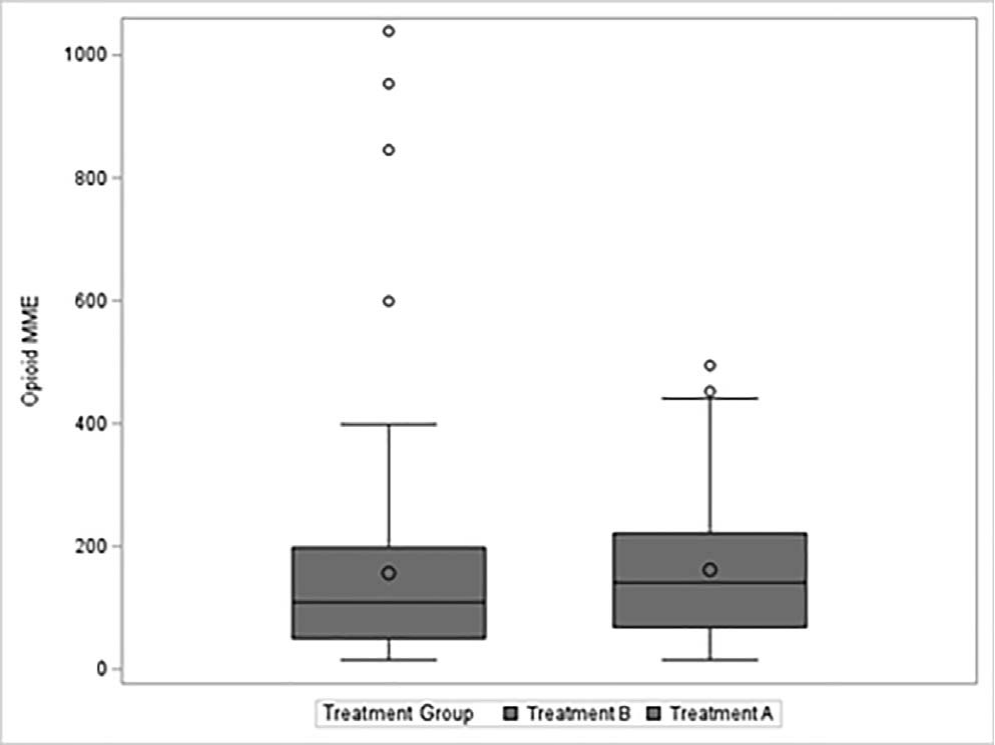
Boxplots of the morphine equivalents used during the hospital stay.

There was no significant difference in average MME between treatment groups during the hospitalization [161.8 mL (SD = 119.3) vs. 156.4 mL (SD = 180.6), *p* = 0.8126] (Table 3). When sub-analyzing by procedure type, patients undergoing THA had a statistically significant reduction in MMEs for those who received LB. That said, this reduction in MMEs could be represented by a single 10 mg dose of oxycodone. Again, there was no difference in morphine equivalents after adjusting for age, sex, and body weight using a linear model (*β* = 0.13, *p* = 0.8206) (Figure 2).

**Figure 3:**
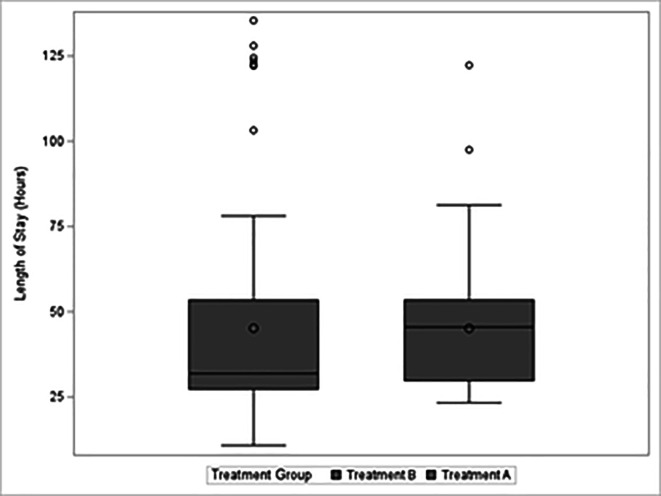
Boxplots of length of stay for Treatments A and B in hours.

The length of inpatient hospital stay was 45.1 h in the Treatment A cohort and 45.2 h in the Treatment B cohort (*p* = 0.9915) (Figure 3).

The cost per patient for a 20 cc vial of LB was $378, and the addition of ropivacaine to each injection was $72.96. In comparison, the cost of a 20 cc vial of non-liposomal 0.5% bupivacaine is $2.17. In a typical PAI where only one local anesthetic is used, using LB increased cost by about $305 per patient when compared to using 0.5% ropivacaine. For context, other hospital cost-saving measures have included restricting the use of vacuum-assisted closure devices unless a patient is deemed exceptionally high risk. The reported cost of such a device is about $94 based on prior literature. Eliminating the use of LB would theoretically allow for the placement of up to three vacuum-assisted closure devices [14].

## Discussion

### Key findings

The key findings of this randomized controlled trial were that the addition of LB did not significantly reduce narcotic consumption in patients undergoing TKA, and the small narcotic reduction it provided in patients undergoing THA likely lacks clinical significance. Potential explanations for this finding include altered uptake of LP near the pericapsular tissues of the hip versus the knee, but currently, this is speculation. Additionally, cost analysis determined that the routine use of LB increased cost by $305 dollars per patient when accounting for the cost of injections as well as intravenous and oral pain medications.

### Comparative literature

Upon FDA approval of LB in 2011, there have been several studies examining the drug’s efficacy in orthopedic populations [15]. While some studies have suggested improved pain control and shortened hospitalizations with the use of LB [16, 17], others have raised questions about its effectiveness [18, 19]. Recently a randomized double-blind study was performed in total knee patients, which demonstrated no improvement in pain management with the addition of LB to the multimodal pain management protocol [20]. Most studies compared the effect of LB with plain bupivacaine in TKA rather than comparing the LB to the standard multimodal injections used by most surgeons. Fewer studies were performed examining the benefit of LB in THA [21–23]. We sought to quantify the benefit of LB added to our multimodal pain management protocol in both total hips and knees. Some studies were funded and performed by the pharmaceutical company that makes LB [8, 24].

The findings presented in this non-industry-funded, randomized, and blinded study demonstrate a statistically significant difference in narcotic usage using morphine equivalents in patients undergoing THA between those who received LB and those who did not. However, there was no difference in narcotic consumption in patients undergoing TKA, nor self-reported pain scores or length of stay. Furthermore, the benefit measured in self-reported pain scores, or length of stay, was minimal, and the difference in narcotic usage between the two THA groups can be represented by a single 10 mg oxycodone dosage. Certainly, in an era of increased scrutiny of narcotic usage, this finding should be considered though it would be difficult to point to a clinically significant difference based only on a 10 mg oxycodone equivalent over the course of hospitalization. Our study adds to the growing body of evidence questioning the value of LB in TJA. Since the initiation of this study, Perets et al. have published a similarly designed study in the THA population with similar results: no benefit to LB over bupivacaine for pain scores, LOS, opioid consumption, and activity up to 72 h after THA in a randomized controlled study of 107 patients [21].

### Clinical implications

Multimodal pain protocols have permitted a push towards less narcotic medications, shorter hospitalizations, and even outpatient total joint surgery. These goals have provided additional motivation to identify PAI cocktails with a prolonged absorption profile. LB is a long-acting, local anesthetic approved for single-dose infiltration into the surgical site with significant local concentrations for up to 96 h [25]. The prolonged absorption of LB leads to a longer duration of action and a slower absorption into the systemic circulation, thereby minimizing serum concentrations [26]. This theoretical longer duration of action has led to widespread regional adoption of LB as a key component of some institutions’ multimodal pain management formularies.

While we found that LB adds very little clinical benefit to standardized multimodal periarticular injections in THA or TKA, there is a significant added cost of $305 dollars per patient, which calculates to a potential hospital savings of over $3M USD per year for 1000 total joint arthroplasties. Based on the results of this study, our institution is no longer using the LB PAI injection.

### Strengths, limitations, and future research

The strengths of this trial include that patients were prospectively randomized and blinded throughout the trial. Additionally, multimodal pain management in the form of a defined protocol was utilized for all patients and managed by an independent and blinded provider using our institution’s standardized arthroplasty postoperative pain management protocol. These strengths allow us to control many variables and isolate the effects of LB’s addition to a widely used PAI rather than a comparison to an inferior injection of merely local anesthetic, as in other studies.

Our study’s limitations are multiple and include the fact that surgeons were unblinded after randomization. We chose to unblind the surgeon after evaluating the potential institutional difficulties and patient risks in maintaining a blinded surgeon. Treatment A remained a relatively opaque injection in Treatment A (with LB) versus transparent liquid in Treatment B. However, a blinded set of nurse practitioners were involved in the day-to-day adjustment of our institution’s multimodal protocol. To maintain the blinded effect, the surgeon and assisting physician assistant were not involved in evaluating or adjusting the pain protocol post-operatively. Another limitation of this study is the difference in the long-acting local anesthetics used in the two joint injection formulations (Table 1). Our institution’s standard PAI utilizes ropivacaine, so we tested LB combined with PAI with bupivacaine (Treatment A) compared to ropivacaine PAI (Treatment B), based upon advice from our pharmacy department regarding mixing ropivacaine and LB. We do not believe this negates the lack of difference between cohorts.

Finally, the use of a spinal block versus general anesthetic could be a confounder affecting narcotic use, specifically in the immediate postoperative period. A spinal anesthetic is our preferred anesthetic per institutional protocol. For a variety of reasons this may not always be possible, and general anesthetic remains as a backup. Our study did not prospectively record the type of anesthesia for each patient and it is possible that the number of patients that received spinal versus general anesthesia could be different between the groups and affect narcotic usage. The study’s randomization should limit the effects of this potential confounder and dissipate its influence between the two cohorts*.* Additionally, the study currently does not have long-term follow-up for pain assessment. However, this provides future opportunities for further research.

## Conclusion

We found that LB adds very little clinical benefit to standardized multimodal periarticular injections in TJA in regard to narcotic usage, pain scores, or LOS, but costs $305 dollars per patient. We believe that in addition to research on the complication profiles and effectiveness of new technologies designed to improve outcomes in TJA, there needs to be cost benefit analysis prior to the wide adoption to control rapidly growing healthcare costs.

## Data Availability

De-identified data will be available upon request for collaboration. Please contact the corresponding author.

## References

[1] Karam JA, Schwenk ES, Parvizi J (2021) An update on multimodal pain management after total joint arthroplasty. J Bone Joint Surg Am 103(17), 1652–1662. 34232932 10.2106/JBJS.19.01423

[2] Halawi MJ, Grant SA, Bolognesi MP (2015) Multimodal analgesia for total joint arthroplasty. Orthopedics 38(7), e616–e625. 26186325 10.3928/01477447-20150701-61

[3] Kelley TC, Adams MJ, Mulliken BD, Dalury DF (2013) Efficacy of multimodal perioperative analgesia protocol with periarticular medication injection in total knee arthroplasty: a randomized, double-blinded study. J Arthroplasty 28(8), 1274–1277. 23608085 10.1016/j.arth.2013.03.008

[4] Ranawat AS, Ranawat CS (2007) Pain management and accelerated rehabilitation for total hip and total knee arthroplasty. J Arthroplasty 22(7 Suppl 3), 12–15. 10.1016/j.arth.2007.05.040 17919586

[5] Mullaji A, Kanna R, Shetty GM, Chavda V, Singh DP (2010) Efficacy of periarticular injection of bupivacaine, fentanyl, and methylprednisolone in total knee arthroplasty: a prospective, randomized trial. J Arthroplasty 25(6), 851–857. 20022457 10.1016/j.arth.2009.09.007

[6] Dasta J, Ramamoorthy S, Patou G, Sinatra R (2012) Bupivacaine liposome injectable suspension compared with bupivacaine HCl for the reduction of opioid burden in the postsurgical setting. Curr Med Res Opin 28(10), 1609–1615. 22900785 10.1185/03007995.2012.721760

[7] Yu SW, Szulc AL, Walton SL, Davidovitch RI, Bosco JA, Iorio R (2016) Liposomal bupivacaine as an adjunct to postoperative pain control in total hip arthroplasty. J Arthroplasty 31(7), 1510–1515. 26872584 10.1016/j.arth.2016.01.004

[8] Dysart SH, Barrington JW, Del Gaizo DJ, Sodhi N, Mont MA (2019) Local infiltration analgesia with liposomal bupivacaine improves early outcomes after total knee arthroplasty: 24-hour data from the PILLAR study. J Arthroplasty 34(5), 882–886-e1. 30799269 10.1016/j.arth.2018.12.026

[9] Navathe AS, Troxel AB, Liao JM, et al. (2017) Cost of joint replacement using bundled payment models. JAMA Intern Med 177(2), 214–222. 28055062 10.1001/jamainternmed.2016.8263

[10] Ji YD, Harris JA, Gibson LE, McKinley SK, Phitayakorn R (2021) The efficacy of liposomal bupivacaine for opioid and pain reduction: a systematic review of randomized clinical trials. J Surg Res 264, 510–533. 33862580 10.1016/j.jss.2021.02.024

[11] Fanelli G, Casati A, Beccaria P, et al. (1998) A double-blind comparison of ropivacaine, bupivacaine, and mepivacaine during sciatic and femoral nerve blockade. Anesth Analg 87(3), 597–600. 9728836 10.1097/00000539-199809000-00019

[12] Kocum A, Turkoz A, Ulger H, Sener M, Arslan G (2007) Ropivacaine 0.25% is as effective as bupivacaine 0.25% in providing surgical anaesthesia for lumbar plexus and sciatic nerve block in high-risk patients: preliminary report. Anaesth Intensive Care 35(4), 510–514. 18020068 10.1177/0310057X0703500408

[13] Ritter MA, Koehler M, Keating EM, Faris PM, Meding JB (1999) Intra-articular morphine and/or bupivacaine after total knee replacement. J Bone Joint Surg Br 81(2), 301–303. 10204938 10.1302/0301-620x.81b2.9110

[14] Kim JJ, Franczyk M, Gottlieb LJ, Song DH (2017) Cost-effective alternative for negative-pressure wound therapy. Plast Reconstr Surg Glob Open 5(2), e1211. 28280658 10.1097/GOX.0000000000001211 PMC5340473

[15] U.S. Food and Drug Administration, Center for Drug Evaluation and Research (2011). EXPAREL NDA 022496 Approval letter.

[16] Asche CV, Dagenais S, Kang A, Ren J, Maurer BT (2019) Impact of liposomal bupivacaine on opioid use, hospital length of stay, discharge status, and hospitalization costs in patients undergoing total hip arthroplasty. J Med Econ 22(12), 1253–1260. 31161837 10.1080/13696998.2019.1627363

[17] Jacob BC, Peasah SK, Shogbon AO, Perlow ER (2017) Postoperative pain management with liposomal bupivacaine in patients undergoing orthopedic knee and hip arthroplasty at a community hospital. Hosp Pharm 52(5), 367–373. 28804154 10.1177/0018578717715382 PMC5551634

[18] Pichler L, Poeran J, Zubizarreta N, et al. (2018) Liposomal bupivacaine does not reduce inpatient opioid prescription or related complications after knee arthroplasty: a database analysis. Anesthesiology 129(4), 689–699. 29787389 10.1097/ALN.0000000000002267 PMC6148397

[19] Chen JJ, Wu YC, Wang JS, Lee CH (2023) Liposomal bupivacaine administration is not superior to traditional periarticular injection for postoperative pain management following total knee arthroplasty: a meta-analysis of randomized controlled trials. J Orthop Surg Res 18(1), 206. 36922892 10.1186/s13018-023-03699-4 PMC10018851

[20] Hamilton TW, Knight R, Stokes JR, et al. (2022) Efficacy of liposomal bupivacaine and bupivacaine hydrochloride vs bupivacaine hydrochloride alone as a periarticular anesthetic for patients undergoing knee replacement: a randomized clinical trial. JAMA Surg 157(6), 481–489. 35385072 10.1001/jamasurg.2022.0713 PMC8988023

[21] Perets I, Walsh JP, Mu BH, et al. (2018) Intraoperative infiltration of liposomal bupivacaine vs bupivacaine hydrochloride for pain management in primary total hip arthroplasty: a prospective randomized trial. J Arthroplasty 33(2), 441–446. 29033152 10.1016/j.arth.2017.09.013

[22] Johnson RL, Amundson AW, Abdel MP, et al. (2017) Continuous posterior lumbar plexus nerve block versus periarticular injection with ropivacaine or liposomal bupivacaine for total hip arthroplasty: a three-arm randomized clinical trial. J Bone Joint Surg Am 99(21), 1836–1845. 29088038 10.2106/JBJS.16.01305

[23] Ma TT, Wang YH, Jiang YF, et al. (2017) Liposomal bupivacaine versus traditional bupivacaine for pain control after total hip arthroplasty: A meta-analysis. Medicine (Baltimore) 96(25), e7190. 28640101 10.1097/MD.0000000000007190 PMC5484209

[24] Bravin LN, Ernest EP, Dietz MJ, Frye BM (2020) Liposomal bupivacaine offers no benefit over ropivacaine for multimodal periarticular injection in total knee arthroplasty. Orthopedics 43(2), 91–96. 31881086 10.3928/01477447-20191223-01

[25] Chahar P, Cummings KC, 3rd (2012) Liposomal bupivacaine: a review of a new bupivacaine formulation. J Pain Res 5, 257–264. 23049275 10.2147/JPR.S27894 PMC3442744

[26] Ostro MJ, Cullis PR (1989) Use of liposomes as injectable-drug delivery systems. Am J Hosp Pharm 46(8), 1576–1587. 2672806

